# Association between SGLT-2 inhibitors and suicide risk in type 2 diabetes and bipolar: a real-world cohort study

**DOI:** 10.3389/fphar.2025.1601118

**Published:** 2025-06-11

**Authors:** En-Wei Chang, Jing-Yang Huang, Shih-Chang Lo, Chien-Ning Huang, Yi-Sun Yang, Edy Kornelius

**Affiliations:** ^1^ School of Medicine, Chung Shan Medical University, Taichung, Taiwan; ^2^ Department of Medical Research, Chung Shan Medical University Hospital, Taichung, Taiwan; ^3^ Department of Internal Medicine, Division of Endocrinology and Metabolism, Chung Shan Medical University Hospital, Taichung, Taiwan; ^4^ Institute of Medicine, Chung Shan Medical University, Taichung, Taiwan

**Keywords:** bipolar, diabetes, SGLT2, DPP4, cohort study

## Abstract

**Background:**

Individuals with bipolar disorder face a significantly elevated suicide risk, and comorbid type 2 diabetes mellitus (T2DM) further complicates their psychiatric and medical outcomes. Sodium–glucose co-transporter-2 inhibitors (SGLT-2i) have demonstrated cardiometabolic benefits in T2DM, but their impact on psychiatric outcomes remains unclear. This study investigates whether SGLT-2i use is associated with a lower risk of suicide-related events compared to dipeptidyl peptidase-4 inhibitors (DPP-4i) in individuals with bipolar disorder and T2DM.

**Methods:**

We conducted a retrospective cohort study using the TriNetX US Collaborative Network, a large electronic health record database. Patients were included if they had bipolar disorder, were receiving active psychiatric treatment, and initiated either SGLT-2i or DPP-4i between 1 January 2015, and 30 June 2024. The primary outcome was the occurrence of suicide-related events, including suicidal ideation, suicide attempt, or intentional self-harm. Secondary outcomes included all-cause mortality, end-stage renal disease (ESRD), diabetic ketoacidosis (DKA), acute kidney injury (AKI), sepsis, genital infections, urinary tract infections (UTIs), and lower-limb amputation. Propensity score matching (1:1) was used to balance baseline characteristics. Cox proportional hazards models estimated hazard ratios (HRs) with 95% confidence intervals (CIs).

**Results:**

The matched cohort included 1,711 patients per group (SGLT-2i vs. DPP-4i). Over a median follow-up of 887 vs. 797 days, suicide-related events occurred in 5.1% of SGLT-2i users vs. 7.3% of DPP-4i users (HR: 0.660, 95% CI: 0.473–0.921, p = 0.0145). All-cause mortality was also lower in the SGLT-2i group (HR: 0.594, 95% CI: 0.451–0.783, p < 0.001). No significant increase in adverse events such as DKA or infections was observed.

**Conclusion:**

In this real-world cohort study, SGLT-2i use was associated with a significantly lower risk of suicide-related events and all-cause mortality compared to DPP-4i in individuals with bipolar disorder and T2DM. Subgroup analyses stratified by age, sex, race, glycemic control (HbA1c), kidney function (eGFR), and baseline lithium use revealed no evidence of increased suicide risk in any subgroup. These findings suggest that SGLT-2 inhibitors may have a neutral to potentially protective effect on suicide-related outcomes. Further prospective studies are warranted to confirm these observations and explore the underlying mechanisms.

## Introduction

Suicide is a leading cause of premature mortality among individuals with bipolar disorder, with risk estimates considerably higher than in the general population (Tondo et al., 2003). Type 2 diabetes mellitus (T2DM) frequently coexists with bipolar disorder, further contributing to morbidity and mortality through metabolic complications and potential psychiatric effects (Calkin et al., 2013; Breznoscakova and Pallayova, 2022). The interplay between mood dysregulation, systemic inflammation, and metabolic dysfunction may increase psychiatric vulnerability in this population, necessitating a deeper understanding of treatment strategies that address both physical and mental health outcomes (Tickell et al., 2022; Chan et al., 2019; Penninx and Lange, 2018).

Sodium–glucose co-transporter-2 inhibitors (SGLT-2i) use among people with type 2 diabetes has grown steadily (Lin et al., 2023). Recent clinical trials and observational studies have highlighted significant cardiometabolic benefits of SGLT-2i in patients with T2DM, including reduced heart failure hospitalizations and slower progression of chronic kidney disease (Zinman et al., 2015; Wiviott et al., 2019; Neal et al., 2017). Some evidence suggests that SGLT-2i may also exert favorable neuropsychiatric effects—potentially through anti-inflammatory mechanisms, improved ketone metabolism, and/or direct central nervous system actions (Tharmaraja et al., 2022; Pawlos et al., 2021). However, their potential role in modulating suicide risk remains unexplored. While the therapeutic advantages of SGLT-2i continue to be elucidated, their impact on patients with mood disorders undergoing active treatment, particularly those with BD, remains poorly characterised. Although evidence-based treatment guidelines for bipolar disorder are well established, evidence concerning the interaction between SGLT2i and mood-stabilising pharmacotherapy are limited, and the potential role of these agents in modulating suicide risk has yet to be explored.

Given the substantial health burden of bipolar disorder and T2DM, understanding whether SGLT-2i influence psychiatric outcomes is critical. Suicide prevention is a major public health priority, and identifying medications that not only improve metabolic health but also reduce psychiatric morbidity could inform safer prescribing practices. This study aims to evaluate whether initiation of SGLT-2i is associated with lower suicide risk in this high-risk population, addressing a gap in the literature and providing clinically relevant insights into the intersection of metabolic and psychiatric health.

## Methods

We conducted a retrospective cohort study using the TriNetX US Collaborative Network, a federated electronic health record database containing de-identified data for ∼90 million patients from 66 healthcare organizations across the United States. Patients were included if they had a diagnosis of bipolar disorder and were receiving treatment for bipolar disorder (any prescription for a mood stabilizer, antipsychotic, or antidepressant) in the year prior to or on the index date. The index date was defined as the first prescription of either an SGLT-2 inhibitor or a DPP-4 inhibitor between 1 January 2015 and 30 June 2024.

Diagnoses were identified using ICD-10-CM codes documented in any clinical encounter setting—whether inpatient, outpatient, or emergency department—and recorded in any diagnosis position, including primary and secondary fields. Medication exposure was determined based on at least one prescription entry with a valid RxNorm code in the patient’s electronic health record, regardless of setting. TriNetX standardizes and harmonizes data through a common data model that incorporates biomedical ontologies from the Unified Medical Language System (UMLS), mapping SNOMED-CT terms to ICD-10-CM and translating drug data from National Drug Codes (NDC) to RxNorm. All definitions for inclusion and exclusion criteria, outcome measures, and baseline covariates—including relevant code sets, encounter types, and diagnostic positions—are detailed in Supplementary Table S1.

DPP-4 inhibitors were chosen as the active comparator group because they represent an alternative second-line glucose-lowering therapy for T2DM, prescribed in a similar clinical context as SGLT-2i. Unlike placebo or no treatment comparisons, using DPP-4i as a comparator helps minimize confounding by indication, as both drug classes are prescribed for similar clinical indications. Additionally, DPP-4 inhibitors have a neutral profile in relation to cardiovascular and renal outcomes, allowing us to better isolate any potential psychiatric effects of SGLT-2i from their known metabolic benefits.

**FIGURE 1:**
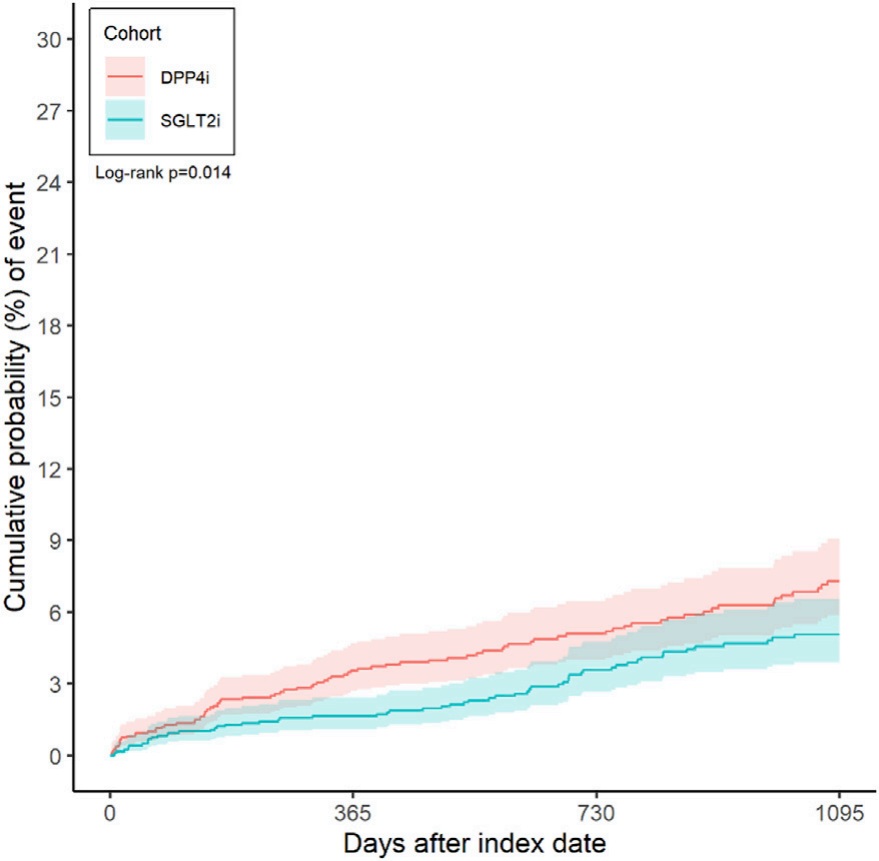
Flow diagram of this study.

We identified 6,538 eligible patients with bipolar disorder who initiated an SGLT-2i or DPP-4i in this timeframe (Figure 1). Patients with any history of suicidal ideation, suicide attempt, or self-harm before index were excluded (n = 4,493) to capture new-onset events. We also excluded patients with end-stage renal disease (ESRD) or dialysis (n = 558) or prior lower-limb amputation (n = 72) before index, as these conditions could significantly alter clinical management, medication eligibility, and overall prognosis. Dialysis patients have unique metabolic and pharmacokinetic considerations that may affect both drug selection and psychiatric outcomes, while prior amputation could indicate severe vascular disease and increase the likelihood of competing risks for mortality. Patients who received both an SGLT-2i and DPP-4i within 1 month after the index date were excluded (n = 560) to avoid exposure misclassification. The remaining cohort consisted of 3,853 patients in the SGLT-2i group and 2,685 in the DPP-4i group.

### Outcomes

The primary outcome was the occurrence of any suicide-related event, defined as a new diagnosis code for suicidal ideation, intentional self-harm, or suicide attempt recorded during follow-up. This composite outcome captured both non-fatal suicidal behaviors and documented ideation. Secondary outcomes included: (1) All-cause mortality; (2) a combined outcome of all-cause mortality or suicide; (3) End-stage renal disease (ESRD), defined by new onset of dialysis or transplant or an ESRD diagnostic code after index; (4) Diabetic ketoacidosis (DKA) episodes; (5) Acute kidney injury (AKI); (6) Sepsis; (7) Genital infections; (8) Urinary tract infections (UTI); and (9) Lower-limb amputations. Each outcome was assessed as a time-to-event from the index date.

The rationale for including secondary outcomes was to capture a negative control with well-known outcomes, ensuring the observed association between SGLT-2i and suicide-related events was not confounded by broader health benefits. By analyzing widely recognized outcomes such as mortality, renal disease, and infections—where SGLT-2 inhibitors have established effects—we aimed to differentiate a specific psychiatric benefit from a general therapeutic advantage of the drug class.

### Propensity score matching (PSM)

To control for confounding, we performed 1:1 PSM between the SGLT-2i and DPP-4i groups. The propensity score was estimated via logistic regression predicting assignment to SGLT-2i versus DPP-4i, using a rich set of baseline covariates. These covariates included patient demographics (age, sex, race, ethnicity, marital status), social and behavioral health factors (e.g., adverse social determinants of health, history of trauma, family history of mental illness, lifestyle problems), psychiatric comorbidities (diagnoses of depression, anxiety disorders, schizophrenia or other psychotic disorders, substance use disorders, sleep disorders, etc.), medical comorbidities (T2DM-related complications, hypertension, dyslipidemia, ischemic heart disease, heart failure, cerebrovascular disease, liver disease, etc.), and prior medications (psychotropic medications such as antidepressants, antipsychotics, mood stabilizers including lithium and anticonvulsants, benzodiazepines, as well as diabetes medications like insulin, metformin, sulfonylureas, etc.). We also included baseline laboratory values where available, categorizing body mass index (BMI), hemoglobin A1c, serum creatinine, estimated glomerular filtration rate (eGFR), and serum lithium levels (for those on lithium therapy). Propensity matching was performed using nearest-neighbor matching without replacement, with a caliper of 0.1 on the propensity score. The matching yielded 1,711 patients in each group (SGLT-2i and DPP-4i) with closely balanced baseline characteristics. Covariate balance was confirmed by standardized mean differences (SMD) for all covariates, with SMD <0.1 indicating negligible residual imbalance; after matching, all covariates had SMD well below 0.1 (most <0.05), suggesting successful balance (Table 1).

**TABLE 1:** Baseline characteristics before and after propensity score matching between SGLT-2i and DPP-4i Groups.

	Before PSM		SMD	After PSM		SMD
	SGLT-2i	DPP-4i		SGLT-2i	DPP-4i	
Total number	3,853	2,685		1711	1711	
Age at index, Mean ± SD	55.3 ± 12.5	57.4 ± 13.0	0.1641	55.3 ± 11.8	55.9 ± 13.3	0.0499
Sex (%)						
Female	2,093 (54.3%)	1,636 (60.9%)	0.1341	996 (58.2%)	1,007 (58.9%)	0.0130
Male	1,611 (41.8%)	973 (36.2%)	0.1144	666 (38.9%)	652 (38.1%)	0.0168
Ethnicity (%)						
Hispanic or Latino	242 (6.3%)	203 (7.6%)	0.0504	127 (7.4%)	131 (7.7%)	0.0089
Not Hispanic or Latino	2,804 (72.8%)	1870 (69.6%)	0.0691	1,212 (70.8%)	1,214 (71.0%)	0.0026
Race (%)						
Asian	65 (1.7%)	62 (2.3%)	0.0445	38 (2.2%)	41 (2.4%)	0.0117
Black	704 (18.3%)	495 (18.4%)	0.0042	308 (18.0%)	311 (18.2%)	0.0046
White	2,563 (66.5%)	1720 (64.1%)	0.0517	1,128 (65.9%)	1,111 (64.9%)	0.0209
Marital status (%)						
Never married	709 (18.4%)	528 (19.7%)	0.0322	323 (18.9%)	360 (21.0%)	0.0541
Divorced	375 (9.7%)	273 (10.2%)	0.0145	175 (10.2%)	174 (10.2%)	0.0019
Widowed	145 (3.8%)	169 (6.3%)	0.1160	73 (4.3%)	95 (5.6%)	0.0595
Adverse socioeconomic determinants of health (%)	210 (5.5%)	103 (3.8%)	0.0768	68 (4.0%)	66 (3.9%)	0.0060
Personal history of psychological trauma (%)	10 (0.3%)	10 (0.4%)	0.0201	10 (0.6%)	10 (0.6%)	0.0000
Family history of mental and behavioral disorders (%)	34 (0.9%)	30 (1.1%)	0.0236	14 (0.8%)	17 (1.0%)	0.0185
Lifestyle-related problems (%)	294 (7.6%)	145 (5.4%)	0.0905	99 (5.8%)	100 (5.8%)	0.0025
Pre-existing medical conditions (%)						
Type 2 diabetes mellitus	2,908 (75.5%)	2,324 (86.6%)	0.2854	1,499 (87.6%)	1,453 (84.9%)	0.0782
Type 2 diabetes mellitus with neurological complications	704 (18.3%)	433 (16.1%)	0.0569	315 (18.4%)	294 (17.2%)	0.0321
Type 2 diabetes mellitus with kidney complications	475 (12.3%)	327 (12.2%)	0.0046	205 (12.0%)	208 (12.2%)	0.0054
Type 2 diabetes mellitus with circulatory complications	243 (6.3%)	120 (4.5%)	0.0815	86 (5.0%)	81 (4.7%)	0.0136
Type 2 diabetes mellitus with ophthalmic complications	130 (3.4%)	80 (3.0%)	0.0225	56 (3.3%)	55 (3.2%)	0.0033
Depression	751 (19.5%)	468 (17.4%)	0.0531	299 (17.5%)	310 (18.1%)	0.0168
Mood disorders, including bipolar disorder	3,853 (100.0%)	2,685 (100.0%)	0.0000	1711 (100.0%)	1711 (100.0%)	0.0000
Anxiety, dissociative, somatoform and other nonpsychotic mental disorders, including posttraumatic stress disorder	1,702 (44.2%)	1,039 (38.7%)	0.1114	703 (41.1%)	701 (41.0%)	0.0024
Schizophrenia, schizotypal, delusional and other non-mood psychotic disorders	418 (10.8%)	478 (17.8%)	0.1995	241 (14.1%)	242 (14.1%)	0.0017
Behavioral disorders, including sleep disorders	187 (4.9%)	94 (3.5%)	0.0676	76 (4.4%)	71 (4.2%)	0.0144
Disorders of adult personality and behavior, including impulse and gender identity disorders	111 (2.9%)	117 (4.4%)	0.0791	65 (3.8%)	61 (3.6%)	0.0124
Symptoms and signs involving an emotional state	82 (2.1%)	77 (2.9%)	0.0474	42 (2.5%)	43 (2.5%)	0.0038
Sleeping disorders including insomnia	1,362 (35.3%)	629 (23.4%)	0.2640	447 (26.1%)	461 (26.9%)	0.0185
Chronic pain	682 (17.7%)	388 (14.5%)	0.0886	265 (15.5%)	283 (16.5%)	0.0287
Alcohol use disorder	254 (6.6%)	142 (5.3%)	0.0552	95 (5.6%)	99 (5.8%)	0.0101
Tobacco use disorder	955 (24.8%)	581 (21.6%)	0.0746	403 (23.6%)	383 (22.4%)	0.0278
Opioid use disorder	108 (2.8%)	55 (2.0%)	0.0491	40 (2.3%)	37 (2.2%)	0.0118
Cannabis use disorder	166 (4.3%)	83 (3.1%)	0.0645	56 (3.3%)	58 (3.4%)	0.0065
Cocaine use disorder	128 (3.3%)	55 (2.0%)	0.0789	35 (2.0%)	37 (2.2%)	0.0081
Other stimulant-related disorders	76 (2.0%)	23 (0.9%)	0.0946	14 (0.8%)	18 (1.1%)	0.0243
Other psychoactive substance-related disorders	149 (3.9%)	77 (2.9%)	0.0554	48 (2.8%)	55 (3.2%)	0.0239
Cancer	435 (11.3%)	247 (9.2%)	0.0690	169 (9.9%)	176 (10.3%)	0.0136
Traumatic brain injury	50 (1.3%)	51 (1.9%)	0.0480	27 (1.6%)	29 (1.7%)	0.0092
Hypertensive diseases	2,784 (72.3%)	1,841 (68.6%)	0.0809	1,169 (68.3%)	1,178 (68.8%)	0.0113
Disorders of lipoprotein metabolism and other lipidemias	2,391 (62.1%)	1,518 (56.5%)	0.1125	995 (58.2%)	1,002 (58.6%)	0.0083
Chronic lower respiratory diseases	1,332 (34.6%)	784 (29.2%)	0.1154	511 (29.9%)	520 (30.4%)	0.0115
Ischemic heart diseases	1,077 (28.0%)	423 (15.8%)	0.2984	276 (16.1%)	300 (17.5%)	0.0375
Heart failure	1,210 (31.4%)	286 (10.7%)	0.5266	188 (11.0%)	232 (13.6%)	0.0784
Cerebrovascular diseases	332 (8.6%)	221 (8.2%)	0.0139	117 (6.8%)	133 (7.8%)	0.0359
Atrial fibrillation and flutter	439 (11.4%)	188 (7.0%)	0.1524	97 (5.7%)	128 (7.5%)	0.0732
Diseases of arteries, arterioles and capillaries	396 (10.3%)	165 (6.1%)	0.1510	100 (5.8%)	115 (6.7%)	0.0361
Other cardiac arrhythmias	308 (8.0%)	112 (4.2%)	0.1604	72 (4.2%)	79 (4.6%)	0.0199
Other sepsis	171 (4.4%)	161 (6.0%)	0.0701	76 (4.4%)	83 (4.9%)	0.0194
Severe sepsis	73 (1.9%)	69 (2.6%)	0.0457	36 (2.1%)	36 (2.1%)	0.0000
Fibrosis and cirrhosis of liver	96 (2.5%)	53 (2.0%)	0.0350	36 (2.1%)	42 (2.5%)	0.0235
Previous medication prescription (%)						
Antidepressants	2,896 (75.2%)	1,944 (72.4%)	0.0628	1,276 (74.6%)	1,277 (74.6%)	0.0013
Antipsychotics	2,523 (65.5%)	1925 (71.7%)	0.1342	1,170 (68.4%)	1,161 (67.9%)	0.0113
Antiepileptics	2,486 (64.5%)	1,785 (66.5%)	0.0412	1,126 (65.8%)	1,140 (66.6%)	0.0173
Benzodiazepine-derivative sedatives or hypnotics	1,689 (43.8%)	1,220 (45.4%)	0.0322	740 (43.3%)	761 (44.5%)	0.0247
Esketamine	10 (0.3%)	10 (0.4%)	0.0201	10 (0.6%)	10 (0.6%)	0.0000
Ketamine	121 (3.1%)	62 (2.3%)	0.0511	48 (2.8%)	48 (2.8%)	0.0000
Lithium (RxNorm: 6,448)	71 (1.8%)	79 (2.9%)	0.0720	42 (2.5%)	43 (2.5%)	0.0038
Lithium (VA: CN750)	108 (2.8%)	112 (4.2%)	0.0746	61 (3.6%)	61 (3.6%)	0.0000
Insulin	71 (1.8%)	79 (2.9%)	0.0720	42 (2.5%)	43 (2.5%)	0.0038
Metformin	1,848 (48.0%)	1,452 (54.1%)	0.1226	919 (53.7%)	902 (52.7%)	0.0199
Alpha glucosidase inhibitors	1,634 (42.4%)	1,481 (55.2%)	0.2572	939 (54.9%)	913 (53.4%)	0.0305
DPP-4 inhibitors	10 (0.3%)	10 (0.4%)	0.0201	10 (0.6%)	10 (0.6%)	0.0000
SGLT2 inhibitors	520 (13.5%)	589 (21.9%)	0.2224	338 (19.8%)	329 (19.2%)	0.0133
Sulfonylureas	117 (3.0%)	100 (3.7%)	0.0381	70 (4.1%)	61 (3.6%)	0.0274
Thiazolidinediones						
BMI, kg/m^2^ (%)						
<30.0	906 (23.5%)	750 (27.9%)	0.1012	440 (25.7%)	425 (24.8%)	0.0202
30–39.9	1,378 (35.8%)	841 (31.3%)	0.0942	575 (33.6%)	567 (33.1%)	0.0099
≥40.0	802 (20.8%)	423 (15.8%)	0.1312	302 (17.7%)	310 (18.1%)	0.0122
Creatinine, mg/dL (%)						
<1.5	2,763 (71.7%)	1732 (64.5%)	0.1550	1,163 (68.0%)	1,152 (67.3%)	0.0137
1.5–2.0	418 (10.8%)	294 (11.0%)	0.0032	163 (9.5%)	163 (9.5%)	0.0000
≥2.0	173 (4.5%)	145 (5.4%)	0.0420	72 (4.2%)	80 (4.7%)	0.0227
HbA1c, % (%)						
<7.0	965 (25.0%)	537 (20.0%)	0.1210	332 (19.4%)	339 (19.8%)	0.0103
7.0–8.9	843 (21.9%)	569 (21.2%)	0.0167	397 (23.2%)	397 (23.2%)	0.0000
≥9.0	727 (18.9%)	455 (16.9%)	0.0502	361 (21.1%)	334 (19.5%)	0.0392
eGFR, CKD-EPI (%)						
<30.0	134 (3.5%)	120 (4.5%)	0.0508	59 (3.4%)	61 (3.6%)	0.0064
30.0–59.9	799 (20.7%)	489 (18.2%)	0.0638	284 (16.6%)	298 (17.4%)	0.0218
≥60.0	2046 (53.1%)	1,264 (47.1%)	0.1207	870 (50.8%)	866 (50.6%)	0.0047
Lithium, mmol/L (%)						
<0.8	97 (2.5%)	77 (2.9%)	0.0216	53 (3.1%)	45 (2.6%)	0.0280
≥0.8	45 (1.2%)	46 (1.7%)	0.0458	27 (1.6%)	23 (1.3%)	0.0195

PSM, propensity score matching; SMD, standardized mean difference; BMI, body mass index; eGFR: estimated glomerular filtration rate.

### Handling of missing data

Several laboratory variables and BMI exhibited partial missingness. After matching, the proportion of missing data was as follows: BMI (33%), serum HbA1c (45%), serum creatinine (29%), estimated glomerular filtration rate [eGFR] (40%), and serum lithium (96%). TriNetX applies an intensive data preprocessing pipeline to harmonize and standardize information across contributing networks. Variables are structured as binary, categorical (converted to binary indicators), or continuous, with missing values for demographics recorded as “unknown.” For clinical and laboratory data, missingness is addressed through presence/absence logic. During matching, we ensured the proportion of missing values was balanced across treatment groups to mitigate bias from incomplete data.

The application of 1:1 PSM resulted in the exclusion of patients who could not be adequately matched across treatment groups—particularly those with distinct clinical profiles that lacked suitable counterparts. This reduction in sample size enhances internal validity by ensuring comparability but may limit generalizability. Specifically, patients who were always treated with either SGLT-2i or DPP-4i and had extreme clinical characteristics were excluded, meaning the study results reflect treatment effects in a well-balanced subset of the population.

### Statistical analysis

We summarized baseline characteristics before and after matching. For outcome analyses, we used Kaplan–Meier methods to estimate cumulative incidence of events at specific time points (3 months, 6 months, 1 year, and 3 years). Incidence rates (IR) per 100 person-years were calculated for each outcome. Cox proportional hazards models were used to compare time-to-event outcomes between the SGLT-2i and DPP-4i groups in the matched cohort, yielding hazard ratios (HR) with 95% confidence intervals. The Cox models were stratified by matched pair to account for the matching. Proportional hazards assumptions were checked and met for the primary outcome. We reported two-sided p-values, considering p < 0.05 as statistically significant. Pre-specified subgroup analyses were conducted for the primary outcome and all-cause mortality, stratifying by key baseline characteristics including age, sex, race, BMI, HbA1c, eGFR, and baseline lithium use. All analyses were performed within the TriNetX Analytics Platform, which performs analyses on aggregated de-identified data. The study protocol was approved by the Institutional Review Board of Chung Shan Medical University Hospital, identified by the reference number CS2-23159.

## Results

### Baseline characteristics

Prior to matching, patients initiating SGLT-2i differed from those initiating DPP-4i on several characteristics. SGLT-2i users were slightly younger on average (mean age 55.3 vs. 57.4 years) and more likely to be male (41.8% vs. 36.2%) than DPP-4i users. Racial and ethnic distributions were similar between groups (66.5% White in SGLT-2i vs. 64.1% in DPP-4i) with small differences (SMD 0.05). All patients by design had bipolar disorder; notably, a large proportion in both groups had co-occurring psychiatric diagnoses such as anxiety disorders (44.2% SGLT-2i vs. 38.7% DPP-4i before match) and many had been on psychotropic medications (75% had prior antidepressant use, 65% antipsychotic use; these rates were comparable between groups after matching). There were some clinical differences: for example, heart failure was more prevalent in the SGLT-2i group (31.4%) than in the DPP-4i group (10.7%) before matching, and SGLT-2i users had higher rates of atherosclerotic cardiovascular disease diagnoses such as ischemic heart disease (28.0% vs. 15.8%). In contrast, schizophrenia spectrum disorders were more common among DPP-4i users (17.8% vs. 10.8%) prior to matching, suggesting that clinicians might have been less likely to prescribe SGLT-2i to patients with severe psychotic disorders. SGLT-2i initiators also had a higher prevalence of obesity-related conditions (e.g., BMI ≥40 in 20.8% vs. 15.8% for DPP-4i) and slightly higher HbA1c on average, reflecting their indication for more intensive glucose lowering.

After PSM, these baseline differences were effectively eliminated. In the matched cohort (n = 1,711 each group), the mean age was ∼55.6 years in both groups (standardized difference 0.05), and 58% were female in each. Racial composition was balanced (e.g., 66% vs. 65% White; 18% vs. 18.8% Black). Comorbid conditions and medication use were also well-matched: for instance, schizophrenia was present in 14.1% of both groups post-match, and the prevalence of heart failure became similar (approximately 12% in SGLT-2i vs. 14% in DPP-4i, SMD ∼0.08). Around three-quarters of patients in each group had been on antidepressants and two-thirds on antipsychotics prior to index, reflecting the high psychiatric treatment needs of the cohort. Likewise, use of mood stabilizers (including lithium and anticonvulsants) and benzodiazepines was comparable between groups post-match. All standardized differences were below 0.1 after matching, indicating a well-balanced cohort (Table 1).

### Primary outcome–Suicide-related events

**FIGURE 2:**
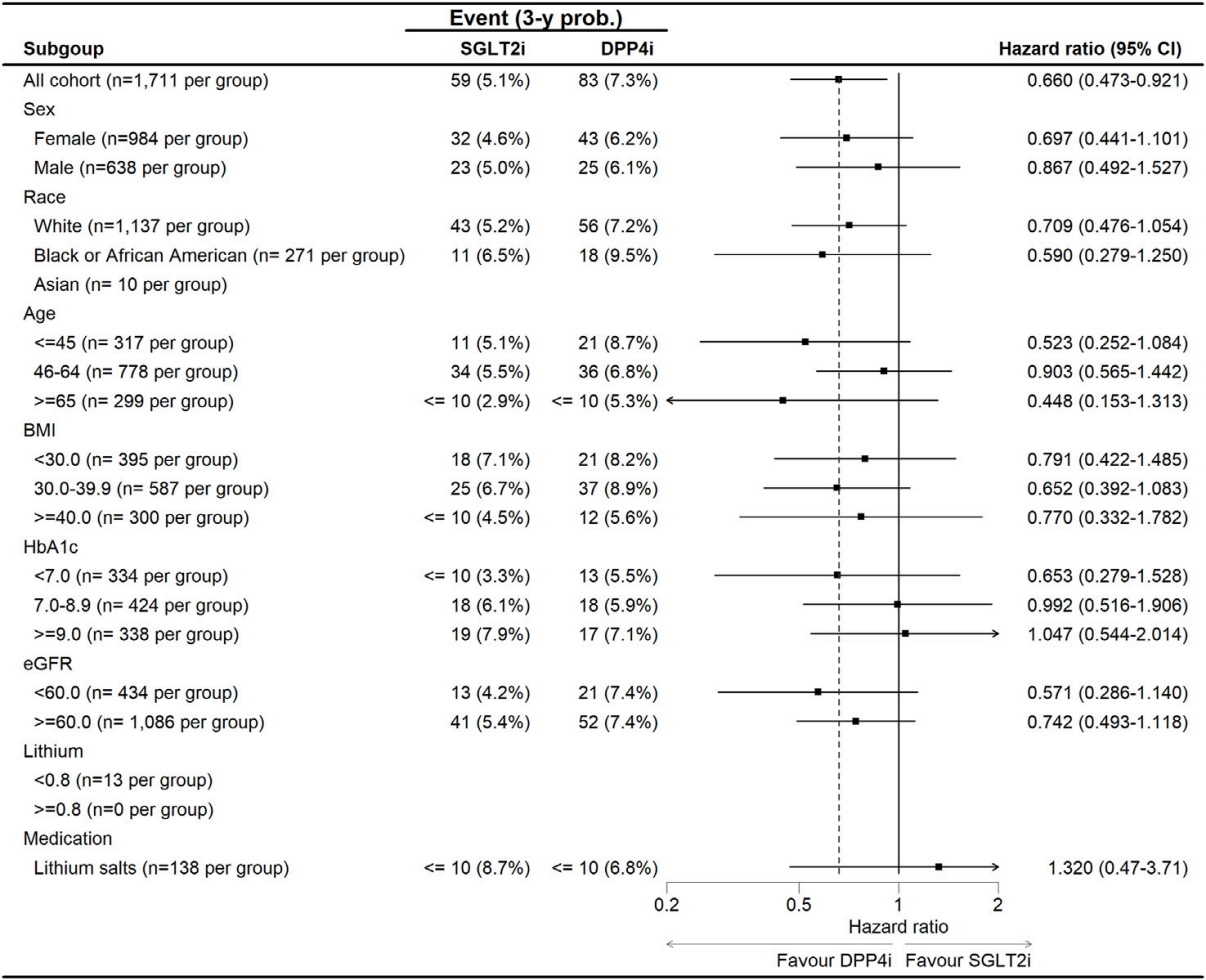
Kaplan-Meier plot for risk of suicide in bipolar patients.

A total of 142 patients experienced a suicide-related event during follow-up in the matched cohort (59 in the SGLT-2i group vs. 83 in the DPP-4i group). The incidence rate of suicide events was 0.17 (95% CI 0.13–0.22) per 100 person-years in the SGLT-2i group and 0.26 (0.21–0.33) per 100 person-years in the DPP-4i group. This corresponded to an incidence rate difference of −0.09 (−0.16 to −0.02) per 100 person-years in favor of SGLT-2i (p = 0.0154). Figure 2 illustrates the cumulative incidence curves for the primary outcome in both groups over time. By 3 months after initiation, the incidence of a suicide event was 0.95% (CI 0.57–1.57) in SGLT-2i users and 1.28% (0.83–1.99) in DPP-4i users. At 6 months, 1.29% (0.83–1.99) of SGLT-2i users had experienced a suicide event versus 2.36% (1.70–3.28) of DPP-4i users. At 1 year, the cumulative incidence was 1.65% (95% CI 1.12–2.43) in the SGLT-2i group compared to 3.48% (2.64–4.58) in the DPP-4i group. At 3 years, the cumulative probability of a suicide-related event was 5.08% (3.92–6.55) with SGLT-2i vs. 7.30% (5.88–9.06) with DPP-4i. In time-to-event analyses, SGLT-2i initiation was associated with a 34% lower hazard of suicide events compared to DPP-4i. The hazard ratio was 0.66 (95% confidence interval 0.473–0.921, p = 0.0145) in favor of SGLT-2i (Table 2).

**TABLE 2:** The risk of Suicide after the index date in propensity score matched cohorts.

	SGLT-2i	DPP-4i	*P* Value
Patients in Cohort, N	1,711	1,711	
Median follow-up, Days (IQR)	887 (729)	797 (843)	
Observed person-years	3,408	3,166	
Events within 3-year, n	59	83	
IR (95% CI)	0.17 (0.13–0.22)	0.26 (0.21–0.33)	
IRD (95% CI)	−0.09 (-0.16 to −0.02)	Reference	0.0154
Rate Ratio (95% CI)	0.660 (0.473–0.922)	Reference	0.0147
Incidence Probability, % (95% CI)			
at 3-month	0.95 (1.57–0.57)	1.28 (1.99–0.83)	
at 6-month	1.29 (1.99–0.83)	2.36 (3.28–1.70)	
at 1-year	1.65 (2.43–1.12)	3.48 (4.58–2.64)	
at 3-year	5.08 (6.55–3.92)	7.30 (9.06–5.88)	
HR (95% CI)	0.660 (0.473–0.921)	Reference	0.0145

IR, incidence rate per 100 person-years.

IRD = IR, difference.

CI, confidence interval.

### Secondary outcomes

SGLT-2i use was associated with significant reductions in several important secondary outcomes (Table 3). Notably, all-cause mortality was substantially lower in the SGLT-2i group. By 3 years, 6.5% of SGLT-2i users had died from any cause, compared to 11.1% of DPP-4i users. The hazard ratio for all-cause mortality was 0.594 (0.451–0.783, p < 0.001), indicating a ∼41% relative risk reduction with SGLT-2i. Similarly, the combined endpoint of “all-cause mortality or suicide” occurred in 11.2% of SGLT-2i patients vs. 17.1% of DPP-4i patients at 3 years, with an HR of 0.63 (0.508–0.782, p < 0.001) in favor of SGLT-2i. These findings reflect the overall survival benefit observed with SGLT-2 inhibitors in this population.

**TABLE 3:** The risk of secondary outcomes after the index date in propensity score matched cohorts.

	Event (3-y cPr)			
	SGLT-2i N = 1711	DPP-4i N = 1711	HR (95% CI)	*P* Value
All-cause mortality and suicide	139 (11.2%)	204 (17.1%)	0.630 (0.508–0.782)	<0.001
All-cause mortality	83 (6.5%)	130 (11.1%)	0.594 (0.451–0.783)	<0.001
Suicide	59 (5.1%)	83 (7.3%)	0.660 (0.473–0.921)	0.015
ESRD	52 (4.2%)	85 (7.4%)	0.570 (0.404–0.805)	0.001
Diabetic ketoacidosis	28 (2.5%)	23 (2.1%)	1.143 (0.659–1.985)	0.634
Acute kidney injury	119 (11.4%)	159 (15.6%)	0.676 (0.533–0.857)	0.001
Sepsis	79 (6.8%)	112 (10.4%)	0.650 (0.488–0.867)	0.003
Genital infections	76 (7.0%)	63 (6.4%)	1.121 (0.803–1.565)	0.502
Urinary Tract Infection	125 (12.4%)	147 (15.0%)	0.744 (0.586–0.944)	0.015
Lower-limb amputations	18 (1.6%)	12 (1.0%)	1.405 (0.677–2.917)	0.359

3-y cPr, 3-year cumulative probability estimated by Kaplan–Meier method; ESRD: end stage renal disease.

Renal outcomes also favored SGLT-2i. The incidence of end-stage renal disease (ESRD) or need for dialysis was 4.2% in the SGLT-2i group vs. 7.4% in the DPP-4i group at 3 years. SGLT-2i initiation was associated with a 43% lower hazard of developing ESRD (HR 0.570, 95% CI 0.404–0.805, p = 0.001). SGLT-2i users also had fewer episodes of acute kidney injury: 11.4% vs. 15.6% (HR 0.676, 0.533–0.857, p = 0.001). The risk of sepsis was significantly lower with SGLT-2i as well, with 6.8% of SGLT-2i users vs. 10.4% of DPP-4i users having a sepsis event (HR 0.650, 0.488–0.867, p = 0.003). These secondary outcomes align with the known systemic benefits of SGLT-2 inhibitors on kidney function and possibly infection-related outcomes.

We also examined outcomes where SGLT-2 inhibitors have known safety considerations. The rate of diabetic ketoacidosis (DKA) was low in both groups and not significantly different: 2.5% in SGLT-2i vs. 2.1% in DPP-4i over 3 years (HR 1.14, 0.659–1.985, p = 0.63). Likewise, the incidence of genital infections was slightly higher in SGLT-2i users (7.0% vs. 6.4%), but this difference was not statistically significant (HR 1.121, 0.803–1.565, p = 0.502). The matched analysis did not show a significant increase in lower-extremity amputations with SGLT-2i: amputation occurred in 1.6% of SGLT-2i patients vs. 1.0% of DPP-4i patients (HR 1.405, 0.677–2.917, p = 0.359), a difference that did not reach significance given the small number of events. Interestingly, urinary tract infections (UTIs) were numerically less frequent in the SGLT-2i group (12.4% vs. 15.0% by 3 years, HR 0.744, 0.586–0.944, p = 0.015). This finding was somewhat unexpected, as SGLT-2i are often thought to modestly increase UTI risk; in our cohort, SGLT-2i users actually had a lower incidence of UTIs than matched DPP-4i users. It is possible that improved glycemic control or other health differences contributed to fewer infections in the SGLT-2i group. Overall, aside from a slight, non-significant excess in genital infections and DKA, we did not observe any safety signal of SGLT-2 inhibitors worsening medical or psychiatric outcomes in bipolar patients. On the contrary, SGLT-2i were associated with broad benefits, including lower mortality and organ failure outcomes, compared to DPP-4 inhibitors.

**FIGURE 3:**
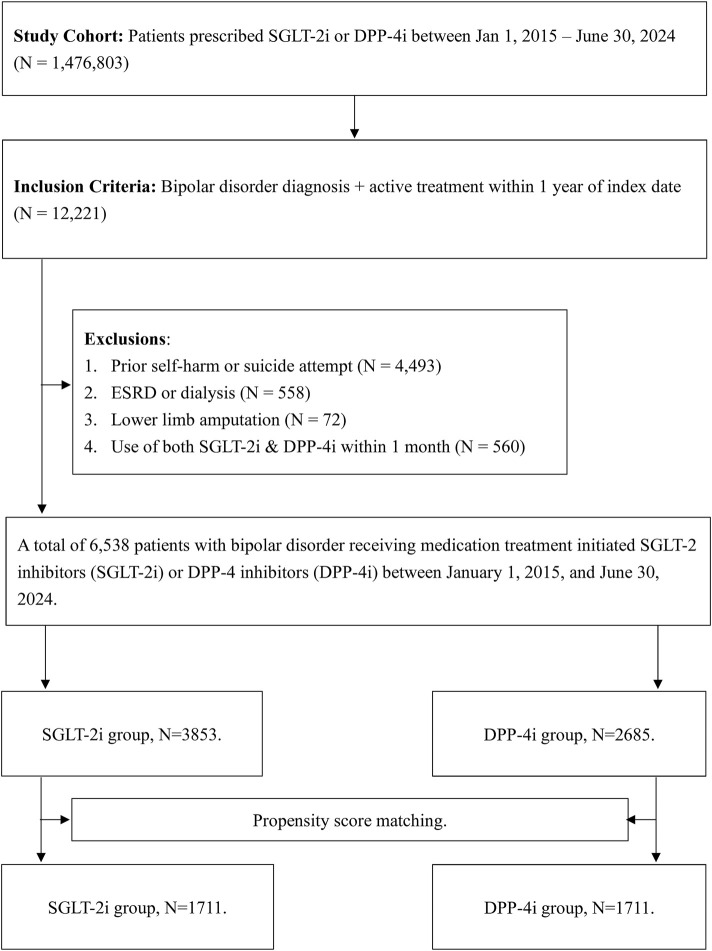
The subgroup analysis for the risk of suicide between SGLT-2i and DPP-4i in patients with bipolar disorders.

Subgroup analysis of the hazard ratios for all-cause mortality and suicide outcomes comparing SGLT-2i versus DPP-4i in patients with bipolar disorder (Figure 3). The forest plot demonstrates a general trend favoring SGLT-2i, with most HR estimates below 1.0, suggesting a reduced risk of suicide and all-cause mortality in this group. However, variability exists across subgroups. For sex, the protective effect of SGLT-2i was stronger in females (HR: 0.697, 95% CI: 0.441–1.101) compared to males (HR: 0.867, 95% CI: 0.492–1.527), though confidence intervals overlapped, suggesting no statistically significant sex-based interaction. Age-stratified analysis revealed the most pronounced reduction in suicide risk for younger patients (≤45 years, HR: 0.523, 95% CI: 0.252–1.084), while risk reduction was more modest in middle-aged (46–64 years, HR: 0.903, 95% CI: 0.565–1.442) and older (≥65 years, HR: 0.448, 95% CI: 0.153–1.313) patients. In racial subgroups, White patients showed a moderate benefit (HR: 0.709, 95% CI: 0.476–1.054), while Black patients had a stronger risk reduction (HR: 0.590, 95% CI: 0.279–1.250). The sample size for Asian patients was too small for reliable interpretation. Patients with HbA1c ≥ 9.0% had a notable protective effect (HR: 0.571, 95% CI: 0.286–1.140), suggesting that those with worse glycemic control might experience greater psychiatric benefits from SGLT-2i. In contrast, for patients with eGFR <60 mL/min/1.73 m^2^, the effect was attenuated (HR: 0.742, 95% CI: 0.493–1.118). Interestingly, patients on lithium salts at baseline showed no significant benefit (HR: 1.320, 95% CI: 0.47–3.71), suggesting a potential interaction between lithium use and the neuropsychiatric effects of SGLT-2i. Overall, no significant interactions were detected (p_interaction >0.05 for all subgroups), indicating that the association between SGLT-2i and reduced suicide risk and mortality was generally consistent across demographic and clinical subgroups.

### Extended follow-up outcomes

To evaluate the long-term association between SGLT-2 inhibitors and both psychiatric and medical outcomes, we conducted follow-up analyses extending to 10 years. Results for suicide-related events, all-cause mortality, and secondary endpoints are presented in Supplementary Tables S2, S3, as well as Supplementary Figure S1. These findings were consistent with our 3-year analysis, reinforcing the durability of observed benefits.

## Discussion

In this large real-world cohort of individuals with type 2 diabetes and bipolar disorder, initiation of SGLT-2 inhibitor therapy was associated with a significantly lower risk of suicide-related events compared to initiation of a DPP-4 inhibitor. To our knowledge, this is the first study to specifically evaluate the relationship between SGLT-2i use and suicidal outcomes in patients with bipolar disorder. The magnitude of 34% risk reduction was substantial, and this finding remained consistent across multiple patient subgroups and was accompanied by parallel reductions in all-cause mortality. These results suggest that, in a population at high risk for both metabolic and psychiatric complications, SGLT-2 inhibitors may confer not only cardiometabolic benefits but also potential protective effects on serious psychiatric outcomes like suicidal behavior.

Our findings align with emerging evidence at the interface of metabolic and psychiatric health. There is a growing recognition that improving metabolic dysfunction can positively impact mood and cognitive disorders (Vasiliu, 2023). Recent clinical data support a psychotropic benefit of SGLT-2i: for instance, a randomized controlled trial reported that adjunctive empagliflozin led to greater improvement in depressive symptoms in patients with major depressive disorder over 8 weeks, compared to placebo (Zandifar et al., 2024). The empagliflozin-treated group showed a significantly larger reduction in depression severity, indicating that SGLT-2i might exert antidepressant effects. Additionally, observational studies have begun to hint at neuropsychiatric advantages of SGLT-2i and related agents. For example, one study suggested that SGLT-2i (and GLP-1 receptor agonists) were associated with lower risks of developing dementia and potentially depression in diabetic patients (Almeida, 2025). While direct evidence in bipolar disorder has been lacking, our results provide a real-world indication that better glycemic management with an SGLT-2i, as opposed to a more weight-neutral DPP-4i, might translate into reduced risk of severe psychiatric deterioration.

Several biological mechanisms could explain why SGLT-2 inhibitors were associated with reduced suicide risk. First, SGLT-2i induce a mild ketogenic state by promoting urinary glucose excretion and shifting metabolism towards fat utilization (Ferrannini et al., 2016). Ketone bodies (such as beta-hydroxybutyrate) readily cross the blood–brain barrier and can serve as an alternative energy substrate for the brain (Hasselbalch et al., 1995). There is evidence that ketosis can have mood-stabilizing and neuroprotective effects (Maalouf et al., 2009); in fact, ketogenic diets have been explored for refractory epilepsy and even mood disorders due to their impact on brain energy metabolism and neurotransmitters (Gasior et al., 2006). SGLT-2i-mediated ketogenesis may similarly exert a stabilizing influence on mood and reduce depressive symptoms, potentially lowering the propensity for suicidal ideation. Second, SGLT-2 inhibitors improve insulin resistance, hyperglycemia, and body weight–factors that have been linked to worse outcomes in bipolar disorder. Poor glycemic control and obesity can contribute to inflammation and endothelial dysfunction (Bakker et al., 2009), which in turn have been implicated in the pathophysiology of mood disorders and suicidality. By ameliorating these metabolic derangements, SGLT-2i might indirectly improve patients’ psychiatric wellbeing. Third, SGLT-2i have anti-inflammatory and neuro-modulatory properties (Heimke et al., 2022). Studies show that SGLT-2 inhibition can reduce markers of systemic inflammation and oxidative stress. Bipolar disorder is often accompanied by chronic inflammation and high oxidative stress, which are thought to negatively affect neurocircuits involved in mood regulation (Andreazza et al., 2008). The use of medications with anti-inflammatory effects (like SGLT-2i) alongside standard treatments could thus help mitigate some biological contributors to suicidal behavior (Zandifar et al., 2024). These hypotheses are supported by the rationale of the above-mentioned trial, which deliberately tested empagliflozin for its potential neuromodulatory benefits. Lastly, patients on SGLT-2i experienced multiple health benefits (fewer cardiovascular and renal events, as seen in our study) (Zinman et al., 2015), which may lead to an overall improvement in quality of life and outlook, possibly reducing suicide risk. It is well established that physical health and mental health are deeply interconnected; improvements in one domain can positively influence the other.

It is also important to address the safety considerations unique to bipolar patients. One theoretical concern has been the interaction between SGLT-2 inhibitors and lithium, a common mood stabilizer. SGLT-2i promote osmotic diuresis due to glucosuria. Since lithium is reabsorbed in the kidneys alongside sodium and water, diuretic effects can lead to enhanced lithium excretion, potentially lowering lithium levels. Indeed, case reports have noted that SGLT-2 inhibitors might reduce serum lithium concentrations and could precipitate relapse in bipolar patients on lithium therapy. In our cohort, a minority of patients (around 3%–4%) were on lithium at baseline. Reassuringly, we did not observe higher psychiatric event rates in SGLT-2i users on lithium; the overall suicide risk was lower in SGLT-2i users regardless of lithium status (Figure 2). This suggests that if SGLT-2i do affect lithium levels, clinicians may have adjusted doses appropriately or the effect was not clinically large in our sample. Nevertheless, this is an important interaction that warrants further study. Clinicians should be mindful to monitor lithium levels when initiating or increasing SGLT-2i in bipolar patients on lithium, to ensure therapeutic levels of lithium are maintained and prevent mood destabilization.

The present study has several strengths. We leveraged a large, multi-center real-world database, which enhances the generalizability of the findings to routine clinical practice. The use of an active comparator (DPP-4 inhibitors) strengthens causal inference by accounting for confounding by indication more effectively than a placebo comparison would; both SGLT-2i and DPP-4i are second-line glucose-lowering therapies, yet they were likely prescribed to somewhat different patient subpopulations, as reflected in baseline differences. By matching on a wide range of psychiatric and medical variables, we achieved a well-balanced comparison that emulates a randomized trial within the constraints of observational data. Notably, all patients in our cohort had a documented bipolar disorder under active treatment, which is a more refined population than just “diabetes patients with a bipolar diagnosis code.” This means our findings specifically pertain to patients with clinically managed bipolar disorder–a group for whom medication management is complex and highly relevant. We were able to examine hard outcomes like suicide attempts and mortality, which, while rare, are critically important in this high-risk population. The consistency of SGLT-2i′s benefit across suicide, mortality, and organ-specific outcomes adds credibility to the findings, as it aligns with known effects of SGLT-2i from randomized trials and extends those benefits to psychiatric outcomes.

Despite these strengths, we acknowledge several limitations. First, as an observational study, we cannot establish definitive causality. There may be residual confounding by factors we could not measure. For example, we did not have direct measures of bipolar disorder severity (e.g., frequency of mood episodes, psychosis severity) or psychosocial factors (such as social support or psychotherapy engagement) that could influence suicide risk. Second, our outcome definition for “suicide-related events” relied on clinical diagnosis codes for suicidal ideation or attempts. This approach has limitations: it may under-capture events, as not all patients disclose suicidal thoughts, and attempts that do not come to medical attention would be missed. Conversely, it might over-capture transient ideation that does not reflect actual suicide attempts. We also could not distinguish completed suicides from non-fatal attempts in the EHR data; deaths are recorded, but cause of death (specifically suicide) is not reliably coded in the database. Thus, our suicide outcome primarily reflects suicidal behavior/ideation rather than confirmed suicide mortality. All-cause mortality was significantly lower with SGLT-2i, and while it is tempting to ascribe some of that to prevention of suicides, we cannot confirm which deaths were due to suicide versus natural causes. Third, the data are dependent on healthcare utilization–if a patient had an outcome but went to a non-network facility, we would not capture it (leading to possible outcome misclassification or censoring). Fourth, our follow-up time, with a maximal of 3 years, may be insufficient to observe very long-term effects on psychiatric outcomes. Bipolar disorder is cyclical, and while a 34% hazard reduction is encouraging, continued observation is needed to ensure this benefit persists beyond 3 years and that no late-emerging risks appear. Fifth, we did not analyze certain potentially relevant outcomes such as effects on mood symptom scales or hospitalizations for mood episodes, as these are not readily available in the EHR-derived dataset. Finally, the use of 1:1 propensity score matching led to the exclusion of patients who could not be adequately matched, such as those with more extreme or treatment-specific clinical profiles. As a result, the study findings are most applicable to a subset of patients with comparable baseline characteristics and may not generalize to all individuals with type 2 diabetes and bipolar disorder.

In summary, this real-world cohort study suggests that among patients with type 2 diabetes and bipolar disorder, SGLT-2 inhibitor therapy is associated with a lower risk of suicide-related events compared to DPP-4 inhibitor therapy. SGLT-2i use was also linked to reduced all-cause mortality and improved renal outcomes in this high-risk population, without a significant increase in adverse events like ketoacidosis or infection. These findings provide preliminary evidence that SGLT-2 inhibitors may be a preferred option for managing T2DM in patients with bipolar disorder, offering metabolic and psychiatric advantages. While observational, our results are hypothesis-generating and support the need for further investigation into the psychiatric safety and benefits of SGLT-2 inhibitors. If confirmed, clinicians can be more confident in using SGLT-2i to improve both physical and mental health outcomes in patients struggling with the dual burden of diabetes and bipolar disorder.

## Data Availability

The datasets presented in this article are not publicly available due to third-party restrictions. This study used data licensed from the TriNetX platform (https://trinetx.com/), which prohibits public redistribution. Access may be requested from TriNetX (join@trinetx.com), subject to data use agreements and possible fees. Further inquiries can be directed to the corresponding author (korn3lius82@gmail.com).

## References

[B1] Almeida O. P. (2025). Risk of depression and dementia among individuals treated with sodium-glucose cotransporter 2 inhibitors and glucagon-like peptide-1 receptor agonists. Curr. Opin. Psychiatry. 10.1097/YCO.0000000000001001 40009763

[B2] Andreazza A. C. Kauer-Sant’Anna M. Frey B. N. Bond D. J. Kapczinski F. Young L. T. (2008). Oxidative stress markers in bipolar disorder: a meta-analysis. J. Affect. Disord. 111 (2-3), 135–144. 10.1016/j.jad.2008.04.013 18539338

[B3] Bakker W. Eringa E. C. Sipkema P. Van Hinsbergh V. W. M. (2009). Endothelial dysfunction and diabetes: roles of hyperglycemia, impaired insulin signaling and obesity. Cell Tissue Res. 335 (1), 165–189. 10.1007/s00441-008-0685-6 18941783

[B4] Breznoscakova D. Pallayova M. (2022). Bipolar disorder and type 2 diabetes mellitus: a bidirectional relationship. Eur. J. Psychiatry 36 (3), 152–162. 10.1016/j.ejpsy.2021.11.002

[B5] Calkin C. V. Gardner D. M. Ransom T. Alda M. (2013). The relationship between bipolar disorder and type 2 diabetes: more than just co-morbid disorders. Ann. Med. 45 (2), 171–181. 10.3109/07853890.2012.687835 22621171

[B6] Chan K. L. Cathomas F. Russo S. J. (2019). Central and peripheral inflammation link metabolic syndrome and major depressive disorder. Physiol. (Bethesda) 34 (2), 123–133. 10.1152/physiol.00047.2018 PMC6586832 30724127

[B7] Ferrannini E. Baldi S. Frascerra S. Astiarraga B. Heise T. Bizzotto R. (2016). Shift to fatty substrate utilization in response to sodium-glucose cotransporter 2 inhibition in subjects without diabetes and patients with type 2 diabetes. Diabetes 65 (5), 1190–1195. 10.2337/db15-1356 26861783

[B8] Gasior M. Rogawski M. A. Hartman A. L. (2006). Neuroprotective and disease-modifying effects of the ketogenic diet. Behav. Pharmacol. 17 (5-6), 431–439. 10.1097/00008877-200609000-00009 16940764 PMC2367001

[B9] Hasselbalch S. G. Knudsen G. M. Jakobsen J. Hageman L. P. Holm S. Paulson O. B. (1995). Blood-brain barrier permeability of glucose and ketone bodies during short-term starvation in humans. Am. J. Physiology-Endocrinol. Metab. 268 (6), E1161–E1166. 10.1152/ajpendo.1995.268.6.E1161 7611392

[B10] Heimke M. Lenz F. Rickert U. Lucius R. Cossais F. (2022). Anti-inflammatory properties of the SGLT2 inhibitor empagliflozin in activated primary microglia. Cells 11 (19), 3107. 10.3390/cells11193107 36231069 PMC9563452

[B11] Lin J. Pearson S. A. Greenfield J. R. Park K. H. Havard A. Brieger D. (2023). Trends in use of sodium-glucose co-transporter 2 inhibitors (SGLT2i) and glucagon-like peptide-1 receptor agonists (GLP-1RA) in Australia in the era of increased evidence of their cardiovascular benefits (2014–2022). Eur. J. Clin. Pharmacol. 79 (9), 1239–1248. 10.1007/s00228-023-03539-8 37449993 PMC10427543

[B12] Maalouf M. Rho J. M. Mattson M. P. (2009). The neuroprotective properties of calorie restriction, the ketogenic diet, and ketone bodies. Brain Res. Rev. 59 (2), 293–315. 10.1016/j.brainresrev.2008.09.002 18845187 PMC2649682

[B13] Neal B. Perkovic V. Mahaffey K. W. de Zeeuw D. Fulcher G. Erondu N. (2017). Canagliflozin and cardiovascular and renal events in type 2 diabetes. N. Engl. J. Med. 377 (7), 644–657. 10.1056/NEJMoa1611925 28605608

[B14] Pawlos A. Broncel M. Woźniak E. Gorzelak-Pabiś P. (2021). Neuroprotective effect of SGLT2 inhibitors. Molecules 26 (23), 7213. 10.3390/molecules26237213 34885795 PMC8659196

[B15] Penninx BWJH Lange S. M. M. (2018). Metabolic syndrome in psychiatric patients: overview, mechanisms, and implications. Dialogues Clin. Neurosci. 20 (1), 63–73. 10.31887/DCNS.2018.20.1/bpenninx 29946213 PMC6016046

[B16] Tharmaraja T. Ho J. S. Y. Sia C. H. Lim N. A. Chong Y. F. Lim A. Y. L. (2022). Sodium-glucose cotransporter 2 inhibitors and neurological disorders: a scoping review. Ther. Adv. Chronic Dis. 13, 20406223221086996. 10.1177/20406223221086996 35432846 PMC9006360

[B17] Tickell A. M. Rohleder C. Ho N. McHugh C. Jones G. Song Y. J. C. (2022). Identifying pathways to early‐onset metabolic dysfunction, insulin resistance and inflammation in young adult inpatients with emerging affective and major mood disorders. Early Interv. Psych. 16 (10), 1121–1129. 10.1111/eip.13260 34852406

[B18] Tondo L. Isacsson G. Baldessarini R. (2003). Suicidal behaviour in bipolar disorder: risk and prevention. CNS Drugs 17 (7), 491–511. 10.2165/00023210-200317070-00003 12751919

[B19] Vasiliu O. (2023). Impact of SGLT2 inhibitors on metabolic status in patients with psychiatric disorders undergoing treatment with second‑generation antipsychotics (Review). Exp. Ther. Med. 25 (3), 125. 10.3892/etm.2023.11824 36845949 PMC9947579

[B20] Wiviott S. D. Raz I. Bonaca M. P. Mosenzon O. Kato E. T. Cahn A. (2019). Dapagliflozin and cardiovascular outcomes in type 2 diabetes. N. Engl. J. Med. 380 (4), 347–357. 10.1056/NEJMoa1812389 30415602

[B21] Zandifar A. Panahi M. Badrfam R. Qorbani M. (2024). Efficacy of empagliflozin as adjunctive therapy to citalopram in major depressive disorder: a randomized double-blind, placebo-controlled clinical trial. BMC Psychiatry 24 (1), 163. 10.1186/s12888-024-05627-0 38408937 PMC10895773

[B22] Zinman B. Wanner C. Lachin J. M. Fitchett D. Bluhmki E. Hantel S. (2015). Empagliflozin, cardiovascular outcomes, and mortality in type 2 diabetes. N. Engl. J. Med. 373 (22), 2117–2128. 10.1056/NEJMoa1504720 26378978

