# Supplementary material for: Association between SGLT-2 inhibitors and suicide risk in type 2 diabetes and bipolar: a real-world cohort study
Source: Front Pharmacol. 2025 Jun 11;16:1601118. doi: 10.3389/fphar.2025.1601118 (PMC12188542; doi:10.3389/fphar.2025.1601118)
Supplement: Supplementary file 1 [file Table1.docx]

**Supplementary Table 1.** Definitions of Inclusion Criteria, Exclusion Criteria, Study Outcomes, and Covariates

| Criteria | Item | Codes | Setting/Position |
| --- | --- | --- | --- |
| Inclusion | SGLT-2i | ATC: A10BK. | First SGLT-2i prescription between Jan 1, 2015, and Jun 30, 2024 (index date defined). |
| Inclusion | DPP-4i | ATC: A10BH. | First DPP-4i prescription between Jan 1, 2015, and Jun 30, 2024 (index date defined). |
| Inclusion | Bipolar disorder | ICD-10-CM: F31. | Diagnosis in any setting, any position, within 6 months on or before the index date. |
| Inclusion | Treatment for bipolar disorder | ATC: N06A (Antidepressants), N03AF (Carboxamide derivatives), N05A (Antipsychotics).  VA: CN750 (Lithium salts). | At least one prescription for any listed drug class within 6 months on or before the index date. |
| Exclusion / Primary Outcome | Suicide-related event | ICD-10-CM: R45.851 (Suicidal ideation), T14.91 (Suicide attempt), Z91.5 (Hx of self-harm), X71–X83 (Intentional self-harm), T36–T50 (Drug poisoning). | Diagnosis in any setting, any position. |
| Exclusion / Outcome | All-cause mortality | Deceased | Any record of death. |
| Exclusion / Outcome | ESRD or dialysis | ICD-10-CM: N18.5, N18.6, R88.0, T82.4, T85.611, T85.621, T85.631, T85.71, Y84.1, Z49, Z91.15, Z99.2; TNX curated: 8001 (eGFR<15); ICD-10-PCS: 5A1D, 3E1M39Z; CPT: 1012740; HCPCS: G0257; SNOMED: 108241001. | ESRD or dialysis in any setting, any position. |
| Exclusion / Outcome | Lower-limb amputation | ICD-10-PCS: 0Y6; CPT: 1005146, 1005298, 1005524. | Procedure code for lower-limb amputation in any setting. |
| Outcome | Diabetic ketoacidosis | ICD-10-CM: E10.1, E11.1, E13.1. | Diagnosis in any setting, any position. |
| Outcome | Acute kidney injury | ICD-10-CM: N17. | Diagnosis in any setting, any position. |
| Outcome | Sepsis | ICD-10-CM: A40, A41, B37.7, A42.7, R65.2. | Diagnosis in any setting, any position. |
| Outcome | Genital infections | ICD-10-CM: B37.3, B37.42, B37.49, N47, N48.1, N76.0-N76.3, N77.1. | Diagnosis in any setting, any position. |
| Outcome | Urinary Tract Infection | ICD-10-CM: N39.0. | Diagnosis in any setting, any position. |
| Covariate | Age at Index | Age at Index |  |
| Covariate | Sex | HL7: F (Female), M (Male). |  |
| Covariate | Ethnicity | HL7: 2135-2 (Hispanic or Latino), 2186-5 (Not Hispanic or Latino). |  |
| Covariate | Race | HL7: 2106-3 (White), 2054-5 (Black or African American), 2028-9 (Asian). |  |
| Covariate | Marital status | Demographics: S (Never married), D (Divorced), W (Widowed). |  |
| Covariate | Adverse socioeconomic determinants of health | ICD-10-CM: Z55-Z65. | For baseline comorbidity, diagnosis recorded in any encounter type and coding position. |
| Covariate | Personal history of psychological trauma | ICD-10-CM: Z91.4. |  |
| Covariate | Family history of mental and behavioral disorders | ICD-10-CM: Z81. |  |
| Covariate | Lifestyle-related problems | ICD-10-CM: Z72. |  |
| Covariate | Type 2 diabetes mellitus | ICD-10-CM: E11. |  |
| Covariate | Type 2 diabetes mellitus with neurological complications | ICD-10-CM: E11.4. |  |
| Covariate | Type 2 diabetes mellitus with kidney complications | ICD-10-CM: E11.2. |  |
| Covariate | Type 2 diabetes mellitus with circulatory complications | ICD-10-CM: E11.5. |  |
| Covariate | Type 2 diabetes mellitus with ophthalmic complications | ICD-10-CM: E11.3. |  |
| Covariate | Depression | ICD-10-CM: F32. |  |
| Covariate | Mood disorders, including bipolar disorder | ICD-10-CM: F30-F39. |  |
| Covariate | Anxiety, dissociative, somatoform and other nonpsychotic mental disorders, including posttraumatic stress disorder | ICD-10-CM: F40-F48. |  |
| Covariate | Schizophrenia, schizotypal, delusional and other non-mood psychotic disorders | ICD-10-CM: F20-F29. |  |
| Covariate | Behavioral disorders, including sleep disorders | ICD-10-CM: F50-F59. |  |
| Covariate | Disorders of adult personality and behavior, including impulse and gender identity disorders | ICD-10-CM: F60-F69. |  |
| Covariate | Symptoms and signs involving an emotional state | ICD-10-CM: R45. |  |
| Covariate | Sleeping disorders including insomnia | ICD-10-CM: G47. |  |
| Covariate | Chronic pain | ICD-10-CM: G89.2. |  |
| Covariate | Alcohol use disorder | ICD-10-CM: F10. |  |
| Covariate | Tobacco use disorder | ICD-10-CM: F17. |  |
| Covariate | Opioid use disorder | ICD-10-CM: F11. |  |
| Covariate | Cannabis use disorder | ICD-10-CM: F12. |  |
| Covariate | Cocaine use disorder | ICD-10-CM: F14. |  |
| Covariate | Other stimulant-related disorders | ICD-10-CM: F15. |  |
| Covariate | Other psychoactive substance-related disorders | ICD-10-CM: F19. |  |
| Covariate | Cancer | ICD-10-CM: C00-D49. |  |
| Covariate | Traumatic brain injury | ICD-10-CM: S06. |  |
| Covariate | Hypertensive diseases | ICD-10-CM: I10-I1A. |  |
| Covariate | Disorders of lipoprotein metabolism and other lipidemias | ICD-10-CM: E78. |  |
| Covariate | Chronic lower respiratory diseases | ICD-10-CM: J40-J4A. |  |
| Covariate | Ischemic heart diseases | ICD-10-CM: I20-I25. |  |
| Covariate | Heart failure | ICD-10-CM: I50. |  |
| Covariate | Cerebrovascular diseases | ICD-10-CM: I60-I69. |  |
| Covariate | Atrial fibrillation and flutter | ICD-10-CM: I48. |  |
| Covariate | Diseases of arteries, arterioles and capillaries | ICD-10-CM: I70-I79. |  |
| Covariate | Other cardiac arrhythmias | ICD-10-CM: I49. |  |
| Covariate | Other sepsis | ICD-10-CM: A41. |  |
| Covariate | Severe sepsis | ICD-10-CM: R65.2. |  |
| Covariate | Fibrosis and cirrhosis of liver | ICD-10-CM: K74. |  |
| Covariate | Antidepressants | ATC: N06A. | For baseline medication, defined as ≥1 prescription record in any encounter type and prescribing position. |
| Covariate | Antipsychotics | ATC: N05A. |  |
| Covariate | Antiepileptics | ATC: N03. |  |
| Covariate | Benzodiazepine-derivative sedatives or hypnotics | VA: CN302. |  |
| Covariate | Esketamine | RxNorm: 2119365. |  |
| Covariate | Ketamine | RxNorm: 6130. |  |
| Covariate | Lithium salts | RxNorm: 6448; VA: CN750. |  |
| Covariate | Insulin | ATC: A10A. |  |
| Covariate | Metformin | RxNorm: 6809. |  |
| Covariate | Alpha glucosidase inhibitors | ATC: A10BF. |  |
| Covariate | Sulfonylureas | ATC: A10BB. |  |
| Covariate | Thiazolidinediones | ATC: A10BG. |  |
| Covariate | BMI | TNX curated: 9083. | Laboratory result recorded in any care setting. |
| Covariate | Serum creatinine | TNX curated: 9024 |  |
| Covariate | Serum HbA1c | TNX curated: 9037 |  |
| Covariate | eGFR | LOINC: 62238-1 |  |
| Covariate | Serum Lithium | TNX curated: LG2034-9 |  |
