# Supplementary figures and images for: Association between SGLT-2 inhibitors and suicide risk in type 2 diabetes and bipolar: a real-world cohort study
Source: Front Pharmacol. 2025 Jun 11;16:1601118. doi: 10.3389/fphar.2025.1601118 (PMC12188542; doi:10.3389/fphar.2025.1601118)

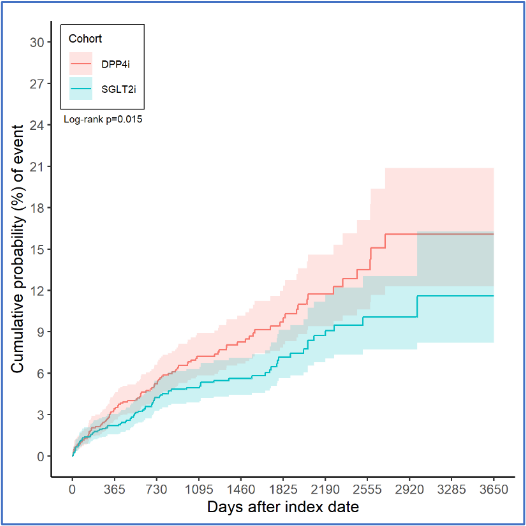

Supplement: Supplementary file 2 [file Image1.tif]
