# Supplementary material for: Association between SGLT-2 inhibitors and suicide risk in type 2 diabetes and bipolar: a real-world cohort study
Source: Front Pharmacol. 2025 Jun 11;16:1601118. doi: 10.3389/fphar.2025.1601118 (PMC12188542; doi:10.3389/fphar.2025.1601118)
Supplement: Supplementary file 3 [file Table2.docx]

**Supplementary Table 2.** The 10-year risk of suicide after the index date in propensity score matched cohorts

|  | SGLT-2i | DPP-4i | *P* value |
| --- | --- | --- | --- |
| Patients in Cohort, N | 1718 | 1718 |  |
| Median follow-up, Days (IQR) | 1002(1318) | 882(1271) |  |
| Observed person-years | 5498 | 4938 |  |
| Events within 3-year, n | 86 | 110 |  |
| IR (95% CI) | 0.16(0.13-0.19) | 0.22(0.18-0.27) |  |
| IRD (95% CI) | -0.07(-0.01 to -0.12) | Reference | 0.016 |
| Rate Ratio (95% CI) | 0.702(0.530-0.931) | Reference | 0.014 |
| Incidence Probability, % (95% CI) |  |  |  |
| at 3-month | 1.32(0.86-2.01) | 1.15(0.72-1.81) |  |
| at 6-month | 1.72(1.18-2.49) | 2.06(1.45-2.92) |  |
| at 1-year | 2.20(1.58-3.07) | 3.35(2.53-4.43) |  |
| at 3-year | 4.95(3.89-6.30) | 7.23(5.85-8.91) |  |
| at 10-year | 11.61(8.22-16.26) | 16.08(12.31-20.86) |  |
| HR (95% CI) | 0.706(0.532-0.936) | Reference | 0.016 |

IR = incidence rate per 100 person-years, IRD = IR difference, CI = confidence interval.
