# Supplementary material for: Association between SGLT-2 inhibitors and suicide risk in type 2 diabetes and bipolar: a real-world cohort study
Source: Front Pharmacol. 2025 Jun 11;16:1601118. doi: 10.3389/fphar.2025.1601118 (PMC12188542; doi:10.3389/fphar.2025.1601118)
Supplement: Supplementary file 4 [file Table3.docx]

**Supplementary Table 3.** The 10-year risk of secondary outcomes after the index date in propensity score matched cohorts

|  | Event (10-y cPr) | |  |  |
| --- | --- | --- | --- | --- |
|  | SGLT-2i  N=1718 | DPP-4i  N=1718 | HR (95% CI) | *P* value |
| All-cause mortality and suicide | 197 (23.3%) | 266 (35.8%) | 0.666(0.554-0.801) | <0.001 |
| All-cause mortality | 120 (14.5%) | 169 (24.4%) | 0.643(0.509-0.813) | <0.001 |
| Suicide | 86 (11.6%) | 110 (16.1%) | 0.706(0.532-0.936) | 0.015 |
| ESRD | 77 (11.0%) | 118 (18.5%) | 0.583(0.438-0.778) | <0.001 |
| Diabetic ketoacidosis | 41 (7.0%) | 37 (7.5%) | 0.996(0.639-1.554) | 0.987 |
| Acute kidney injury | 183 (29.0%) | 225 (39.9%) | 0.692(0.570-0.842) | <0.001 |
| Sepsis | 131 (22.7%) | 174 (27.2%) | 0.674(0.537-0.846) | 0.001 |
| Genital infections | 129 (19.2%) | 86 (12.4%) | 1.394(1.061-1.832) | 0.016 |
| Urinary Tract Infection | 181 (28.6%) | 200 (39.5%) | 0.762(0.623-0.932) | 0.008 |
| Lower-limb amputations | 28 (4.2%) | 15 (1.8%) | 1.711(0.913-3.204) | 0.090 |

10-y cPr, 10-year cumulative probability estimated by Kaplan–Meier method; ESRD: End stage renal disease
